# Plasma and Muscle Myostatin in Relation to Type 2 Diabetes

**DOI:** 10.1371/journal.pone.0037236

**Published:** 2012-05-16

**Authors:** Claus Brandt, Anders R. Nielsen, Christian P. Fischer, Jakob Hansen, Bente K. Pedersen, Peter Plomgaard

**Affiliations:** Centre of Inflammation and Metabolism, Department of Infectious Diseases and CMRC, Rigshospitalet, Faculty of Health Sciences, University of Copenhagen, Copenhagen, Denmark; University of Las Palmas de Gran Canaria, Spain

## Abstract

**Objective:**

Myostatin is a secreted growth factor expressed in skeletal muscle tissue, which negatively regulates skeletal muscle mass. Recent animal studies suggest a role for myostatin in insulin resistance. We evaluated the possible metabolic role of myostatin in patients with type 2 diabetes and healthy controls.

**Design:**

76 patients with type 2 diabetes and 92 control subjects were included in the study. They were matched for age, gender and BMI. Plasma samples and biopsies from the vastus lateralis muscle were obtained to assess plasma myostatin and expression of myostatin in skeletal muscle.

**Results:**

Patients with type 2 diabetes had higher fasting glucose (8.9 versus 5.1 mmol/L, P<0.001), plasma insulin (68.2 versus 47.2 pmol/L, P<0.002) and HOMA2-IR (1.6 versus 0.9, P<0.0001) when compared to controls. Patients with type 2 diabetes had 1.4 (P<0.01) higher levels of muscle myostatin mRNA content than the control subjects. Plasma myostatin concentrations did not differ between patients with type 2 diabetes and controls. In healthy controls, muscle myostatin mRNA correlated with HOMA2-IR (r = 0.30, P<0.01), plasma IL-6 (r = 0.34, P<0.05) and VO2 max (r = −0.26, P<0.05), however, no correlations were observed in patients with type 2 diabetes.

**Conclusions:**

This study supports the idea that myostatin may have a negative effect on metabolism. However, the metabolic effect of myostatin appears to be overruled by other factors in patients with type 2 diabetes.

## Introduction

Human myostatin was first cloned in 1998 [1]. Myostatin, or growth/differentiation factor 8 (GDF-8), belongs to the transforming growth factor-β (TGF-β) superfamily and has been identified as a major regulator of muscle mass [2]. Myostatin is a peptide hormone produced by skeletal muscle and secreted into the circulation. Myostatin is a negative regulator of muscle mass and is well preserved across species as judged from its expression in fish, birds, cows and humans [3].

Interestingly, recent observations in animal models suggest that myostatin is involved in the regulation of energy metabolism as hypermuscular myostatin knock-out mice have reduced fat mass and are protected from dietary-induced insulin resistance [4]–[6]. Furthermore, animal models have suggested a role for myostatin in diabetic muscle atrophy as ob/ob diabetic mice have higher levels of myostatin expression, and reduced muscle mass as well as fiber cross-sectional area [7], [8]. *In vitro* studies of myostatins effect on glucose metabolism is contradictory as myostatin was shown to inhibit glucose uptake in a placental cell line [9] however an increased glucose uptake has also been demonstrated using human placenta extracts [10]. The finding that myostatin knock-out mice are protected against obesity-induced insulin resistance as measured by a hyperinsulemeamic clamp [5] suggests an effect of myostatin on insulin-mediated glucose uptake. Furthermore, myostatin knock-out mice show an increased AMP-activated protein kinase activity in skeletal muscle, which could explain the increased insulin sensitivity [11]. In contrast, myostatin has been shown to increase AMP-activated protein kinase activity in C2C12 myotubes thereby improving glucose uptake [12]. Taken together *in vitro* and animal studies suggests that myostatin affects glucose uptake, but the literature is not consistent. An inhibitory association is supported by a gene expression study in which an transcriptomic array revealed an increased myostatin expression in skeletal muscles of patients with type 2 diabetes [13]. Although loss of muscle mass is a clear clinical feature of type 2 diabetes [14], [15], it is uncertain whether increased circulating myostatin plays a role in the metabolic deterioration of skeletal muscle in individuals with obesity and insulin resistance.

To elucidate the associations between myostatin and insulin resistance, lean body mass, fitness and low-grade inflammation, we evaluated circulating levels of myostatin as well as skeletal muscle expression of myostatin in patients with type 2 diabetes and in controls, who were closely matched for gender and body mass index (BMI).

## Materials and Methods

### Study design

A cross-sectional design was employed. As previously described, subjects (n = 233) were recruited by advertising in a local newspaper. They received oral and written information about the experimental procedures before giving their written, informed consent to participate. Assessment of the type 2 diabetes diagnosis was based on information from each subject and confirmed by an oral glucose tolerance test (OGTT). Thirty-four subjects were excluded as they were classified to have an impaired glucose tolerance (IGT) [16], [17]. From 168 subjects (92 healthy controls and 76 patients with type 2 diabetes) sufficient sample material was available for analysis of myostatin. In brief, participants were screened to isolate such metabolic conditions other than type 2 diabetes, which are known to influence body composition and the immune system. Exclusion criteria were treatment with insulin, recent or ongoing infection, a history of malignant disease and known dementia. Participants reported to the laboratory between 8 and 10 am after an overnight fast. They did not take any medication in the 24 h preceding the examination, and the type 2 diabetics did not take their oral anti-diabetic medication for 1 week preceding the examination. A general health examination was performed. Blood samples were drawn from an antecubital vein and a biopsy was obtained from the vastus lateralis muscle. An oral glucose tolerance test (OGTT) was performed on the same day.

The study was approved by the Ethics Committee of the Copenhagen and Frederiksberg Communities (KF 01-141/04).

### OGTT

Blood samples were drawn before and 1 and 2 h after the participant had drunk 500 ml of water containing 75 g of dissolved glucose. The WHO diagnostic criteria were applied. Participants found to have IGT were excluded from the study.

### Fitness test

Cardiorespiratory fitness was measured by the Åstrand-Rhyming indirect test of maximal oxygen uptake [18].

### Body composition

Bone mass density (BMD), whole body fat and fat-free tissue masses, trunk and extremities were measured using DXA scanning (Lunar Prodigy Advance; GE Medical Systems Lunar, Milwaukee, WI). DXA scanning does not distinguish between subcutaneous and intraabdominal fat located in the trunk region. Software (Prodigy, enCORE 2004, version 8.8, GE Lunar Corp., Madison, WI) was used to estimate the mass of regional and total fat and fat-free tissue.

### Plasma samples

Blood samples were drawn into glass tubes containing EDTA, which were immediately spun at 3500 g for 15 min at 4°C. Plasma was isolated and stored at −20°C until analysed.

### Tissue samples

Skeletal muscle biopsies were obtained from vastus lateralis using a Bergström biopsy needle [26]. The biopsies were immediately frozen in liquid nitrogen and stored at −80°C until analysed.

### Plasma analysis

The plasma myostatin assay is a competitive immunoassay. The standards and samples are pre-incubated with a polyclonal rabbit-anti human recombinant myostatin (full length) antibody. During this pre-incubation free myostatin is bound by the myostatin-antibody. The pre-incubated samples and standards are then transferred to a microtiterplate coated with human recombinant myostatin (full length). The unbound antibodies bind to the immobilized antigen on the microtiterplate. By use of a peroxidase conjugated goat-anti-rabbit antibody the bound antibody is detected. Tetramethylbenzidine (TMB) is used as a peroxidase substrate. Finally, an acidic stop solution is added to terminate the reaction, whereby the colour changes from blue to yellow. The intensity of the yellow colour is inversely proportional to the concentration of myostatin. A dose response curve of the absorbance unit (optical density, OD at 450 nm) vs. concentration is generated using the values obtained from the standard. Myostatin in the samples is determined from this curve. Detection limit: 0.273 ng/ml. Inter assay CV: <15% Intra assay CV: <10%. Immundiagnostik AG, Bensheim, Germany, conducted the plasma myostatin measurements. Plasma concentrations of TNF-α and IL-6 were measured by ELISA (R&D Systems, Minneapolis, MN, USA). Samples were analysed in duplicate and mean concentrations were calculated. In plasma, levels of cholesterol (HDL and LDL), triglycerides, C-reactive protein (CRP), glucose and insulin were measured using routine laboratory methods. Based on the fasting plasma concentrations of glucose and insulin, the level of insulin resistance was calculated using the homeostasis model assessment of insulin resistance, version 2 (HOMA2-IR) of 1998 (software available at http://www.dtu.ox.ac.uk/) [19].

### RNA isolation, reverse transcription and real-time PCR

Total RNA was extracted from ∼40 mg muscle tissue using Trizol Reagent (Invitrogen, Carlsbad, CA, USA) following the manufacturer's instructions. In summary, muscle tissue was homogenized in 1 ml Trizol Reagent for 15 s using a Qiagen Tissuelyser (Qiagen Nordic, Copenhagen, Denmark). Chloroform was added and the phases were separated by centrifugation. The aqueous phase with the RNA was transferred to a fresh tube and the RNA precipitated by adding isopropanol and left at −20°C for 1 h. After another centrifugation, the RNA pellet was washed in 75% ethanol and finally dissolved in 50 µl diethylpyrocarbonate-treated water.

The RNA concentration was determined spectrophotometrically and 2 µg total RNA was reversed-transcribed in a total volume of 100 µl using the Taqman Reverse Transcription Kit (Applied Biosystems, NJ, USA) and random hexamers as primers. Real-time PCR was performed using an ABI 7900 Sequence Detection System (Applied Biosystems). The mRNAs for myostatin and the endogenous control, β-actin, were amplified using predeveloped assays (Applied Biosystems). The PCR conditions followed the procedure recommended by the manufacturer, with 10 µl reaction volume and each sample run in triplicate for 50 cycles. The mRNA content of both the target and the endogenous control gene was calculated from the cycle threshold values by using a standard curve constructed from a serial dilution of aliquots of cDNA pooled from all the samples.

### Statistical analysis

Data are generally presented as means with confidence interval of the mean. If the data were not normally distributed, a logarithmic transformation was applied and the data were presented as geometric means. Logarithmic transformation was performed on all data except: age, BMI, BMD, lean body mass, fat mass and LDL cholesterol. For comparisons between the groups (control versus type 2 diabetes and low versus high myostatin) a t-test was used for continuous variable whereas a χ^2^ test was used for categorical variables. Analysis for correlations was performed using Pearson's approach. A multiple regression analysis was done using a general linear model (PROC GLM). All analyses were performed using SAS software version 9.1 (SAS institute, Cary, NC, USA). P<0.05 was considered significant.

## Results

### Characterization of control subjects and patients with type 2 diabetes

Seventy-six patients with type 2 diabetes and 92 control subjects were investigated in the study. The patients with diabetes were slightly older than the control subjects, but gender distribution was similar in the two groups, Table 1. BMI and fat mass as determined by DXA scan were similar in both groups. Fasting glucose, plasma insulin, and HOMA2-IR levels were higher, whereas plasma total cholesterol, LDL cholesterol, HDL cholesterol levels were lower in the patients with type 2 diabetes than in the control subjects. Plasma triglycerides, TNF-α and IL-6 were higher in the patients with diabetes than in the control subjects; these differences remained significant after age and gender adjustment, Table 1.

**Table 1:** Subject characteristics.

	Healthy control subjects (n = 92)	Type 2 diabetes patients (n = 76)	P-value	P*-value
***Clinical:***				
**Age (years)**	53.2 (50.7–55.7)	58.2 (55.7–60.7)	0.006	----
**Gender (M/F)**	64/28	57/19	0.43	----
**BMI (kg/m^2^)**	30.0 (28.7–31.4)	30.6 (29.2–31.9)	0.57	0.026
**VO_2 max_ (L/kg)**	28.7 (26.8–30.7)	23.3 (21.6–25.4)	0.0001	0.0004
***Body composition:***				
**Bone mass density**	3.0 (2.9–3.1)	2.8 (2.7–2.9)	0.03	0.002
**Lean body mass**	58.6 (55.9–61.3)	57.9 (55.1–60.7)	0.73	0.034
**Fat mass**	30.5 (27.3–33.6)	29.3 (26.9–31.7)	0.55	0.033
***Glycemic parameters:***				
**Fasting glucose (mmol/L)**	5.1 (5.0–5.2)	8.9 (8.2–9.8)	<0.0001	<0.0001
**Fasting insulin (pmol/L)**	47.2 (40.8–54.5)	68.2 (56.7–82.1)	0.002	<0.0001
**HOMA2-IR**	0.9 (0.8–1.0)	1.6 (1.3–1.9)	<0.0001	<0.0001
***Lipids***				
**Plasma cholesterol (mmol/L)**	5.3 (5.1–5.5)	4.8 (4.5–5.1)	0.009	0.0008
**LDL cholesterol (mmol/L)**	3.5 (3.4–3.7)	2.9 (2.7–3.2)	<0.0001	<0.0001
**HDL cholesterol (mmol/L)**	1.5 (1.4–1.6)	1.3 (1.2–1.4)	0.006	<0.0001
**Plasma triglycerides (mmol/L)**	1.2 (1.0–1.3)	1.5 (1.3–1.8)	0.01	0.0011
***Inflammation:***				
**CRP (mg/L)**	2.4 (2.0–2.9)	3.0 (2.5–3.7)	0.08	0.006
**TNF-α (ng/L)**	2.4 (2.3–2.5)	2.7 (2.5–2.8)	0.005	0.0045
**IL-6 (ng/L)**	1.2 (1.1–1.5)	1.7 (1.4–2.0)	0.02	0.0023
**IL-18 (ng/L)**	224 (207–243)	242 (221–264)	0.20	0.0315

Body composition, glycaemic variables, plasma lipids, and inflammatory markers in healthy control subjects and type 2 diabetes patients. BMI; Body mass index, P indicates a significant difference between the groups, P* is corrected for age and gender. P<0.05 is considered significant.

### Myostatin levels are increased in patients with type 2 diabetes

**Figure 1:**
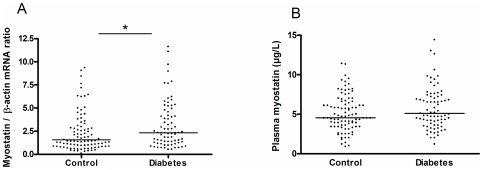
Skeletal muscle mRNA content (A) and plasma myostatin (B) in healthy control (n = 92) and patients with type 2 diabetes (n = 76). Individual data are presented and the bar indicates the geometric mean. * indicates a significant difference between healthy controls and patients with type 2 diabetes, P<0.05.

Skeletal muscle myostatin mRNA content was 1.4 fold (P<0.05) higher in patients with type 2 diabetes when compared to the control group, Figure 1A. This difference remained significant after adjustment for age and gender (P<0.001). The plasma myostatin concentration was slightly elevated in patients with type 2 diabetes 5.1 (4.6–5.7) µg/L compared to 4.5 (4.1–5.0) µg/L in control subjects. However this difference was only significantly different when correcting for age and gender (P = 0.0261), Figure 1B. When the data from the patients with diabetes and the control subjects were combined, plasma and muscle myostatin levels were similar in men and women (P = 0.5 and P = 0.2 respectively).

### Associations of muscle myostatin mRNA content and plasma myostatin with clinical, glycaemic, lipid, and inflammatory variables

**Figure 2:**
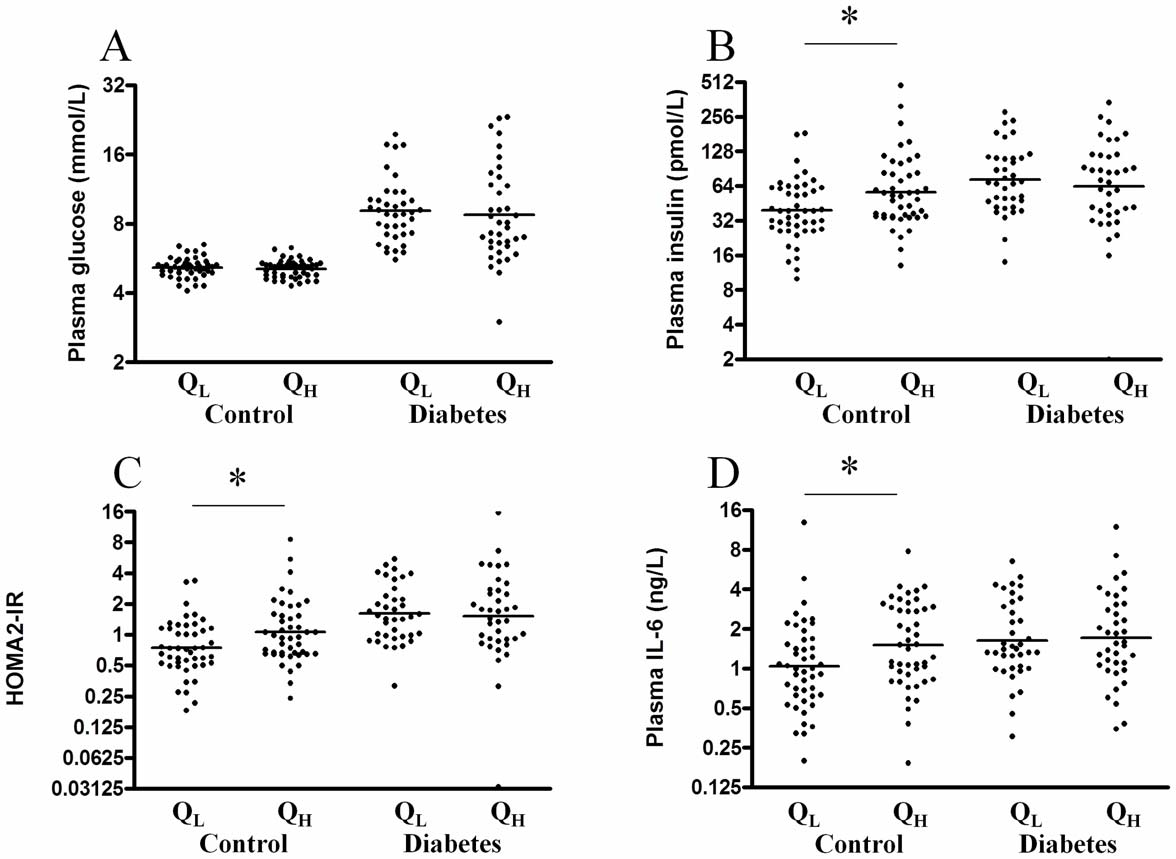
Muscle myostatin mRNA divided in low (Q_L_) and high (Q_H_) content in control subjects and patients with type 2 diabetes, respectively. The bar represents geometric means for plasma (A), plasma insulin (B), HOMA2-IR (C) and plasma IL-6 (D). * indicates a difference between low versus high muscle content of myostatin mRNA. P<0.05 is considered significant.

**Figure 3:**
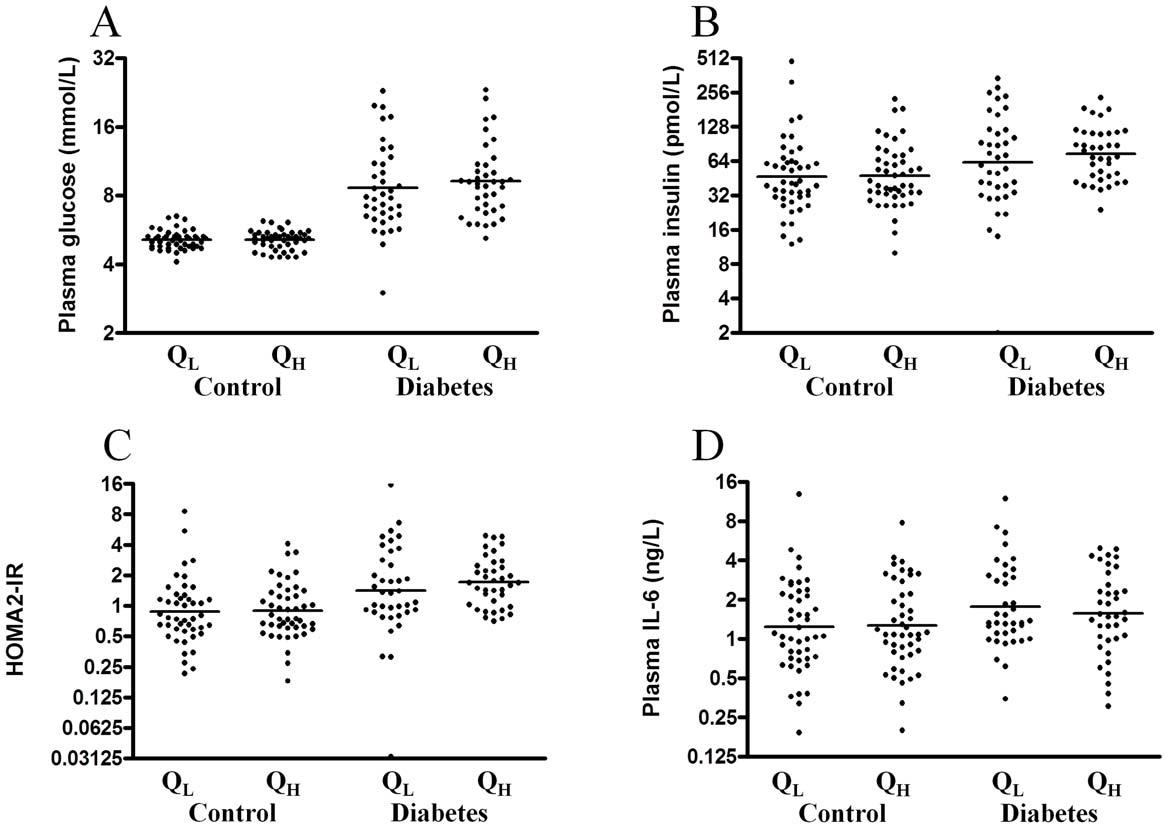
Plasma myostatin divided in low (Q_L_) and high (Q_H_) content in control subjects and patients with type 2 diabetes, respectively. The bar represents geometric means for plasma (A), plasma insulin (B), HOMA2-IR (C) and plasma IL-6 (D). * indicates a difference between low versus high muscle content of myostatin mRNA.

To evaluate the association between clinical and biochemical markers of insulin resistance Pearson's correlations were performed and appear from Table 2. The skeletal muscle content of myostatin mRNA correlated positively with fasting insulin, HOMA2-IR, plasma IL-6, CRP, BMI and triglycerides, and negatively with maximal oxygen uptake (VO2 max) when all participants were analysed together. Only fasting blood glucose correlated with plasma myostatin and only when the healthy controls and patients with type 2 diabetes were analysed in combination, Table 2. However when the groups were analysed separately, muscle myostatin mRNA content only correlated significantly in the control group. Plasma myostatin and muscle myostatin mRNA content was positively correlated in the healthy controls only. The healthy controls and patients with type 2 diabetes were divided into low (QL) and high (QH) muscle content of myostatin mRNA and the fasting glucose, insulin, HOMA2-IR, and plasma IL-6 levels were compared. In the patients with type 2 diabetes no difference was observed. However, in the healthy controls with a high myostatin mRNA content in the vastus muscle, a higher level of fasting insulin, HOMA2-IR and plasma IL-6 could be demonstrated, Figure 2. No differences were found when the same analysis was performed for circulating myostatin, Figure 3.

**Table 2:** Pearson's correlations to plasma myostatin and myostatin mRNA expression in skeletal muscle tissue.

	Control subjects (n = 92)		Patients with type 2 diabetes (n = 76)		Combined (n = 168)	
Variable	Plasma	Muscle	Plasma	Muscle	Plasma	Muscle
***Clinical:***						
**Age (years)**	0.04	−0.26*	0.15	0.01	0.14	−0.10
**BMI (kg/m^2^)**	−0.06	0.31*	−0.03	−0.02	−0.04	0.19*
**VO_2_ max (L O_2_/kg)**	0.09	−0.26*	0.03	−0.08	0.03	−0.23*
***Body composition:***						
**Bone mass density**	0.07	−0.07	0.19	0.18	0.08	−0.02
**Lean body mass**	0.08	0.16	0.09	0.15	0.08	0.14
**Fat mass**	−0.09	0.29**	0.03	−0.14	−0.06	0.13
***Glycemic parameters:***						
**Fasting glucose (mmol/L)**	0.02	−0.04	0.19	−0.02	0.17*	0.15
**Fasting insulin (pmol/L)**	−0.02	0.31**	−0.02	−0.08	0.005	0.18*
**HOMA2-IR**	−0.03	0.30**	0.04	−0.05	0.05	0.20**
***Lipids***						
**Plasma cholesterol (mmol/L)**	−0.09	−0.10	0.08	0.16	−0.03	−0.02
**LDL cholesterol (mmol/L)**	−0.09	−0.16	−0.03	0.09	−0.10	−0.12
**HDL cholesterol (mmol/L)**	−0.11	−0.17	−0.05	0.06	−0.10	−0.10
**Plasma triglycerides (mmol/L)**	0.09	0.24*	0.09	−0.02	0.11	0.15*
***Inflammation:***						
**CRP (mg/L)**	−0.06	0.22*	−0.10	0.07	−0.06	0.19*
**TNF-α (ng/L)**	−0.08	0.23*	0.07	−0.15	0.02	0.11
**IL-6 (ng/L)**	−0.002	0.34*	−0.13	0.15	−0.03	0.29**
**IL-18 (ng/L)**	0.07	0.13	0.001	−0.03	0.05	0.08
***Myostatin***						
**Plasma myostatin (µg/L)**		0.21*		−0.01	—	0.14
**Muscle myostatin mRNA content**	0.21*		−0.01		0.14	—

Pearson's correlations coefficients *r* between plasma myostatin and muscle myostatin mRNA, respectively, and different clinical and biochemical variable.

* P<0.05;

** P<0.01.

### Multivariate analysis

To further investigate the relationship between the variables found to correlate with muscle myostatin mRNA content, a multivariate analysis was performed. Besides diabetes, age, and gender, only predictors that correlated significantly were included. As it appears from Table 3, age and plasma IL-6 remained significant. Plasma myostatin was solely significantly correlated with fasting glucose.

**Table 3:** Multivariate analysis, including variables that were found to correlate with muscle myostatin mRNA content.

	Muscle myostatin mRNA.	
Over all model:	P = 0.0011 n = 168	
Explanatory variables	Estimate	P-value
Diabetes	0.127	0.05
Age	−0.007	0.03
Gender	0.097	0.15
BMI	−0.003	0.65
VO_2_ max	−0.460	0.09
HOMA2-IR	0.038	0.74
TAG	0.014	0.90
CRP	−0.033	0.10
IL-6	0.216	0.04

## Discussion

The present study demonstrates that skeletal muscle myostatin mRNA is elevated in patients with type 2 diabetes when compared to healthy control subjects. Furthermore we show that muscle myostatin mRNA content is associated with impaired insulin sensitivity, increased triglycerides, and low-grade chronic inflammation as well as obesity and a poor fitness level. Interestingly, clear associations were found in healthy controls, but were absent in type 2 diabetes patients. Therefore, if a causal relationship exists between myostatin and metabolism, it appears that the negative, regulatory effects of myostatin on metabolism are overruled by other factors in advanced type 2 diabetes. In accordance, a positive association between plasma and muscle myostatin was only observed in the healthy controls, which may suggest an alteration in the regulatory mechanism with diabetes. It appears that plasma myostatin, compared to muscle myostatin, was a less strong marker of metabolism, as plasma myostatin was only associated with fasting glucose.

Very few studies have assessed the plasma levels of circulating myostatin in humans. Lakshman et al [20] applied an in house developed ELISA to measure serum myostatin in 50 young and 48 old men and found serum concentrations at 8.0 and 7.0 µg/L, respectively. In the present study, the average plasma level of myostatin was 4.8 µg/L. The circulating levels are within the same range; however the discrepancy could be due to the differences in matrix (plasma versus serum) and differences in populations, as well as to the large range of variation observed between individuals. In the present study, no difference was detected between young and old, which most likely was due to the low number (n = 7) of young participants (age<35 years). Even though no differences were observed between young and old, a negative association with age and muscle myostatin mRNA content was observed, which remained significant when adjusting for insulin resistance, inflammatory status and fitness. Lakshman et al did not find an association between lean body mass and circulating myostatin, which is in line with the present study, where no correlation was found between lean body mass and neither plasma myostatin, nor muscle myostatin mRNA content.

Myostatin KO mice demonstrate improved insulin sensitivity [5], [6], suggesting that myostatin is involved in glucose regulation. However, these mice concomitantly had altered adiposity, but interestingly treating ob/ob mice with anti-myostatin antibodies resulted in an improved glucose clearance, without any changes in fat mass [21]. The effects of myostatin on glucose metabolism could be due to effects on the muscle tissue itself, as only inhibition of myostatin signaling in skeletal muscle and not adipose reveal an improved insulin sensitivity [6]. An alternative mechanism could be via TNF-α [5], which is known to cause insulin resistance [22]. Interestingly, a positive association was observed between circulating TNF-α and myostatin mRNA expression in the control subjects, supporting the observations made in mice. In the multivariate analysis muscle myostatin mRNA content was predicted by age and plasma IL-6, when adjusting for insulin resistance, plasma triglycerides and obesity. It is noteworthy that plasma IL-6 and fitness are inversely related [23], [24]. A reduction in myostatin mRNA with improved fitness is in line with a reduction in muscle myostatin mRNA content after an acute bout of exercise [25], however this inverse association in the present data is not significant if adjusted for BMI. Very few studies have evaluated the response of plasma myostatin in humans in relation to exercise or training, whereas several have demonstrated a reduction of muscle mRNA content [25]–[28]. One study reported that after 10 weeks of resistance training, circulating levels of myostatin have decreased by approximately 20% [29]. The present cross-sectional data suggest that plasma myostatin is a poor marker of fitness, although this does not rule out the possibility that individual changes in plasma myostatin could be a valuable marker. Furthermore these human data reveal a positive association between insulin resistance and myostatin mRNA expression in the skeletal muscle in healthy subjects. Increased myostatin mRNA expression might be a predisposing marker for the development of insulin resistance in healthy subjects. Interestingly, the fitness level correlated inversely with the myostatin mRNA only in the group of healthy subjects, why it could be speculated that an increased insulin resistance, which is associated with increased myostatin can be counter acted by exercise.

Myostatin is involved in adipocyte differentiation [30] and recently, Hittel et al [31] compared 6 lean (BMI<25) with 9 extremely obese (BMI>40) subjects using western blotting and found an association with muscle and plasma myostatin to both BMI and HOMA2-IR. In the present study a positive association was also observed regarding muscle myostatin mRNA and both BMI and insulin resistance as measured by HOMA in normal controls subject. However, the present data contribute by allowing adjustment for age, inflammation and fitness, which reveals that the association with HOMA2-IR and BMI was no longer significant.

In conclusion, high muscular expression of myostatin is associated to impaired metabolism, systemic inflammation, obesity and poor fitness level in healthy subjects. These associations are disrupted in patients with type 2 diabetes, where no associations are observed although myostatin mRNA levels are moderately enhanced. The findings of the present study as well as data from recent experimental reports make us suggest that muscle-produced myostatin exerts direct and negative effects on glucose and lipid metabolism. However, the metabolic effect of myostatin appears to be overruled by other factors in full-blown type 2 diabetes.
